# Untangling the Threads: The Impact of Co-Occurring OCD and ADHD Symptoms for Black and/or Latiné Youth

**DOI:** 10.3390/children12060674

**Published:** 2025-05-24

**Authors:** Terumi S. Randle, Laurel N. Miskovic, Victoria R. Grant O’Daniel, Anjo I. Okechukwu, Najiya Shahzad, Kayla C. Mkandawire, Madeline Warrick, Zoe R. Smith

**Affiliations:** Department of Psychology, Loyola University Chicago, Chicago, IL 60660, USA; trandle2@luc.edu (T.S.R.); lmiskovic@luc.edu (L.N.M.); vgrant2@luc.edu (V.R.G.O.); aokechukwu@luc.edu (A.I.O.); nshahzad@luc.edu (N.S.); kmkandawire@luc.edu (K.C.M.); mwarrick@luc.edu (M.W.)

**Keywords:** ADHD, OCD, depression, anxiety, trauma, multicultural youth

## Abstract

**Introduction:** Obsessive-compulsive disorder (OCD) and attention-deficit/hyperactivity disorder (ADHD) are neuropsychiatric diagnoses that commonly co-occur, as approximately 25% of youth with OCD also have a diagnosis of ADHD and 11% of youth with ADHD have OCD. Individuals with ADHD and OCD are also commonly treated for symptoms of depression, anxiety, and traumatic experiences. Conversely, Black and Latiné youth in the United States have limited access to culturally responsive providers to address these conditions due to systemic racism; the lower rates of appropriate diagnosis, treatment, and inclusion in research create worsening symptoms of OCD. Thus, we sought to understand how ADHD symptoms affect OCD symptoms and how these comorbid diagnoses, in addition to anxiety disorders, affect reported anxiety, depression, and trauma for Black and/or Latiné teens. **Procedures:** Participants (*N* = 48) are Black and/or Latina/é/o youth with ADHD in the United States. Self and parent-report measures were completed for ADHD, anxiety, and depression, and a semi-structured interview was conducted to assess current mental health diagnoses (OCD, ADHD, trauma). **Results:** A path analysis showed higher levels of ADHD inattentive (ADHDI) symptoms (β = 0.34) were positively associated with obsessions. In contrast, higher ADHD hyperactive/impulsivity symptoms (ADHDHI) (β = −0.11) were negatively associated with obsessions. Neither ADHDI nor ADHDHI symptoms were associated with compulsions. Interestingly, ADHDI (β = 0.33) & obsessions (β = 0.28) were both associated with depression; however, ADHDHI was negatively associated with depression (β = −0.29). Importantly, ADHDI was associated with trauma (β = 0.13) and obsessions were strongly associated with anxiety (β = 0.38). **Conclusions:** These findings may allow for better screenings and treatments for co-occurring OCD and ADHD symptoms and a greater understanding of the impact depression, anxiety, and trauma have on neurodivergent Black and/or Latiné youth.

## 1. Introduction

Obsessive-compulsive disorder (OCD) and attention-deficit hyperactivity disorder (ADHD) are neuropsychiatric diagnoses that commonly co-occur, as approximately 25–33% of youth with OCD also have a diagnosis of ADHD [1]. Inversely, among youth with an ADHD diagnosis, 8–11% of individuals meet criteria for OCD, which is notably higher than the population prevalence at 2–3% [2]. OCD is an internalizing condition that is characterized by obsessions, recurring, intrusive thoughts, and compulsions, repetitive behaviors performed to reduce the discomfort of obsessive thoughts. ADHD is a neurodevelopmental condition characterized by inattention, hyperactivity, and impulsivity [3,4]. Both are heterogeneous disorders that vary in symptom presentations [5,6].

The general prevalence of the conditions over the lifetime has significant differences and similarities. Within the US, ADHD is estimated to be prevalent among 10.47% of children and adolescents aged 4 to 17 [3]. The worldwide prevalence of ADHD in children is 5.29% [7]. In addition, ADHD is a condition that typically manifests during childhood around age 12 for 5% and at least 4% of adults report their ADHD symptoms remained in adulthood [8,9]. In comparison, OCD has a lifetime prevalence of 1.6–2.3% and onsets typically around young adulthood at the age of 19 [10,11]. However, more evidence suggests that pediatric OCD symptoms occur and lead to increased adult OCD prevalence and severity later in life [12,13]. Moreover, there is a suggestion that symptoms for either condition that manifests in childhood might lead to an increased chance of comorbidity for both ADHD and OCD.

Youth with ADHD and OCD often exhibit overlapping symptoms that can complicate diagnosis and treatment. Both conditions can involve impulsivity, inattention, and difficulty with executive functioning, while OCD specifically presents with intrusive thoughts and compulsive behaviors. The overlap occurs when ADHD-related impulsivity and inattention intersect with OCD’s compulsions and obsessional thoughts, leading to challenges in differentiating the disorders. Research indicates that the comorbidity of ADHD and OCD in children and adolescents may exacerbate symptoms, making it essential for clinicians to carefully assess and consider both conditions to create an effective, individualized treatment plan [1]. Early identification and tailored interventions are key to managing the complex interaction between ADHD and OCD in youth.

### 1.1. Compulsivity-Impulsivity Dimension

The association between ADHD and OCD might be explained through the compulsive-impulsive dimension. This theory conceptualizes a continuum where facets of compulsivity and impulsivity are spread across a diametrically intricate spectrum and both are present in conditions that would traditionally be considered only compulsive or impulsive disorders [14,15]. Impulsivity is defined by the actions that are risky and/or prematurely expressed, whereas compulsivity is defined by actions that are habitual, unpleasantly repetitive acts to prevent perceived negative consequences [14,15,16].

The literature remains indecisive about the relationship between OCD and impulsivity. Certain studies found that there are elevated elements of impulsivity present in OCD features such as aggressive obsessions, checking, and risky decision making [15,16,17,18]. Other findings have disputed this and maintain that OCD has few impulsivity factors and that clinical OCD populations endorse less impulsive behaviors than nonclinical populations [19,20]. Frydman et al. (2020) propose that the connection between OCD and impulsivity could potentially be subjective rather than objective, as impulsivity measured through self-reports was more associated with severe OCD symptoms than impulsivity measured through neurocognitive measures [21].

Moreover, the relationship between ADHD and compulsivity is likewise inconclusive. There is evidence that children with ADHD impulsively hoard items and over time the hoarding compulsion becomes a strategy to combat impairment such as disorganization and losing items [22]. In addition, adults with childhood ADHD were more likely to endorse OCD symptoms than adults without ADHD or adult-onset ADHD impairment [14,23]. These findings all suggest that while the current literature is inconclusive, there is a possibility of understanding OCD and ADHD through the continuum theory. Despite this, no conclusive evidence has emerged.

### 1.2. ADHD, OCD, and Anxiety Symptoms

Anxiety disorders, which frequently co-occur with both ADHD and OCD, manifest as excessive worry or fear. In youth, the comorbidity of these disorders is common, with research indicating that many youth with ADHD also experience symptoms of OCD, anxiety, or depression, which can significantly impact their daily functioning [24]. These diagnoses share similar features, such as difficulty concentrating or irritability. People with both OCD and generalized anxiety disorder (GAD) exhibit more severe obsessions and compulsions, particularly in aggressive, sexual/religious, and contamination dimensions [25]. Anxiety disorders comorbid with ADHD are also associated with exacerbated attention difficulties, disruptive social behaviors, negative affectivity, and slowed reaction times [26,27].

There is a demonstrated comorbidity of anxiety disorders with OCD in clinical populations. Anxiety is characterized by excessive worry or fear, apprehensive expectations, and physiological changes such as fatigue, restlessness, and muscle tension [28]. OCD was once classified as an anxiety disorder, the significance of the clinical and biological distinctiveness of OCD has led to the inclusion of obsessive-compulsive and related disorders (OCRDs) as a new category in the DSM-V [25,28]. Despite this change, the comorbidity of OCD and anxiety disorders, particularly with GAD, is recognized as an association with implications for approaching clinical mental health care.

The high rates of comorbidity between OCD and anxiety disorders are consistently reported across previous clinical studies. OCD is noted to have a high prevalence with anxiety disorders such as social anxiety disorder, panic disorder and GAD [29]. Along with this higher prevalence, the comorbidity of these disorders leads to more severe clinical outcomes and OCD symptoms. The co-occurrence is linked to OCD symptoms having an earlier onset, being more severe, and having a lengthier duration. Likewise, the symptoms are associated with higher levels of anxiety, higher rates of suicidal ideation and behavior, increased avoidance behaviors, and a greater number of specific anxiety and mood disorders [25,29]. Research indicates that individuals with both OCD and anxiety disorders, such as GAD, exhibit more severe obsessions and compulsions, particularly in aggressive, sexual/religious, and contamination dimensions [25]. The significance of this prevalence and effect on symptomology emphasizes the need for integrated treatment approaches. The implication that anxiety disorders such as GAD amplify the severity of OCD symptoms makes the future of treatment options more complex and challenging.

Despite these insights, there is a significant gap in the literature regarding the comorbidity of OCD and anxiety with Black and Latiné youth. While there is a demonstrated effect of cross-cultural influence on OCD symptoms, most research focuses on general populations with limited exploration of how these conditions impact youth who hold systemically oppressed racial and ethnic identities. This gap underscores the need for studies that address the experiences and challenges faced by Black and Latiné youth with comorbid OCD and anxiety. With the high comorbidity of OCD and other anxiety disorders, it can lead to a poorer prognosis due to greater severity of both disorders and higher rates of distress and suicidality [30]. OCD and anxiety can feed off one another, each one worsening the other. Despite OCD’s low lifetime prevalence at 2–3%, it has been found to have a high correlation with other anxiety disorders such as social phobia, panic disorder, and GAD; with a lifetime prevalence of 23.4%, 23.1%, and 18.3% [2,31]. The high rates of comorbidity imply a high amount of overlap in symptoms in both disorders, symptoms which are largely known for lowering quality of life. This provides a compelling reason to investigate the interaction of OCD with other anxiety disorders to try and see what impact this interaction can have on youth.

The prevalence of anxiety disorders in individuals with ADHD is significantly higher than in the general population, with a comorbid prevalence rate of 3.9–84% compared to the rate of the general population with 4.3–47.1% [32]. Adults with childhood ADHD have more severe anxiety than adults without childhood ADHD [33]. For youth, 5% to 15% reported experiencing significant anxiety that co-occurs with their ADHD [26,27]. The prevalence of comorbidity with these conditions necessitates the understanding of overlapping symptomology.

ADHD and anxiety commonly co-occur and present with overlapping symptoms; this co-occurrence complicates assessment, diagnosis, and treatment. It is suggested that anxiety can inhibit hyperactive and impulsive behaviors in children with ADHD; this suppression potentially explains why youth with comorbid ADHD and anxiety have delayed clinical presentations [27]. Anxiety disorders comorbid with ADHD are also associated with exacerbated attentional difficulties, disruptive social behaviors, negative affectivity, and slowed reaction times [26,27]. Anxiety may also contribute to physical health issues like sleep disturbances and gastrointestinal problems. On a neurological level, slowed information processing in ADHD can increase anxiety, as individuals struggle to meet social and academic norms [27].

### 1.3. ADHD, OCD, and Depression

The overlap between ADHD, OCD, anxiety, and depression in youth can lead to a more complex clinical presentation. For example, a child with ADHD may exhibit impulsive behaviors that are interpreted as compulsions in the context of OCD, or symptoms of anxiety may manifest as restlessness or distractibility, which are typically associated with ADHD. Additionally, depression can exacerbate ADHD symptoms by increasing a child’s irritability, decreasing their motivation, and worsening attention difficulties. When these disorders co-occur, it can lead to a cycle of negative reinforcement, where one disorder exacerbates the symptoms of another, leading to greater impairment in social, academic, and emotional functioning [34]. This complicates the process of providing effective treatment, as clinicians must carefully evaluate the interactions between these disorders.

Racial discrimination is a common source of stress for Black and/or Latiné youth; the more racial discrimination experienced, the higher the levels of obsessive-compulsive symptoms over time—however racial identity works as a protective factor [35]. This suggests that Black and Latiné with OCD are at a higher risk of depression in part because of racism, but, with protective factors such as strong positive racial identity beliefs and pride, it buffers the relationships.

### 1.4. Trauma Exposure

Youth with ADHD and OCD who experience trauma often face compounded challenges in managing their symptoms, as trauma can exacerbate both diagnoses. ADHD, characterized by inattention, impulsivity, and hyperactivity, may be further intensified in a child who has experienced traumatic events, as they may struggle with heightened emotional responses and difficulty regulating behavior. Trauma and ADHD share similar traits including deficits in emotion regulation and executive functioning, contributing to misdiagnoses [36]. OCD, which involves intrusive thoughts and compulsive behaviors, may become more pronounced in response to trauma, as children may engage in compulsions as a means of coping with feelings of anxiety or fear related to traumatic experience. Research indicates that trauma can disrupt emotion regulation and increase the severity of symptoms in children with ADHD and OCD, often leading to a cycle of maladaptive coping strategies [37]. Youth with co-occurring PTSD and OCD experienced a greater severity of symptoms, including an increased number of intrusive distress and fears as well as experiencing less control in respect to rituals [38]. The symptomology of PTSD and OCD often encompasses both intrusive images representing past events and avoiding upsetting stimuli, yet OCD is unique in its manifestations of intrusive thoughts and implementation of rituals [38].

Currently, no studies explore the interaction of comorbid ADHD, OCD, anxiety, and trauma symptoms, which our study aims to address. Viewing this association between ADHD, OCD, and trauma through an intersectional lens may highlight even greater impacts on health, specifically for Black and/or Latina/é/o youth.

### 1.5. ADHD, OCD, and Black and/or Latiné Youth

There is limited work that examines the effects of ADHD and OCD symptoms for Black and/or Latiné youth. The prevalence of ADHD for Black and Latiné youth is generally lower than the prevalence reported for White youth [4]. At age 12, White youth have a 10.6% prevalence of receiving an ADHD diagnosis compared with 8.1% for Black youth and 5.9% for Latiné youth. The prevalence of OCD for Black adults is reported as 1.6% [39] and the reported prevalence of OCD for Latiné people is varied as they are represented as less than 1% of the participants in clinical trials focused on OCD [6]. It should also be noted that many studies conducted to understand OCD for Black and Latiné people do not consider the entire diaspora. Due to this, this could affect clinical responsiveness and consideration of identity for Black and/or Latiné youth outside of the United States. This difficulty in understanding the prevalence of OCD and ADHD symptoms adds to the challenge of assisting Black and/or Latiné youth in clinical spaces.

In addition, there are noted cultural differences in OCD symptomatology for obsessions and compulsions. Compared to White participants, Black participants reported higher rates of contamination/cleaning rituals and hoarding [5,40]; whereas Latiné participants reported higher rates of contamination and religious scrupulosity [6]. Given the cultural differences in how obsessions and compulsions are performed for Black and Latiné individuals, more extensive OCD research must be provided to understand culturally related factors [5]. Although ADHD and OCD are heterogeneous conditions, the inadequate research focused on Black and Latiné youth diminishes reported experiences of either condition alone or co-occurring. It also constrains information about how these conditions interact with secondary psychological conditions. 88–95.6% of Black individuals with OCD experience at least one other comorbid disorder, including major depressive disorder (MDD), GAD, and post-traumatic stress disorder (PTSD; [39,41]. There is limited information about how Latiné people are affected by comorbidities with OCD symptoms, but generally, it reflects the comorbidities previously explored. In addition, many individuals with OCD report experiencing physical ailments such as physical pain, which mirrors the psychosomatic symptoms reported by Latiné people instead of OCD symptoms [40,42].

Cultural differences have been found in the types of obsessions and compulsions experienced, such as unique relationships between beliefs and cleaning and checking behaviors, as well as morality and thought control [40]. For example, literature suggests that Black youth experience differing symptomology may be influenced by family expectations of obedience and respect, affecting adults’ accommodation of OCD related behaviors in their children [43]. This authoritarian parenting is often needed to keep Black youth safe in a world that is racist against them but unfortunately can worsen symptoms or make youth feel invalidated. Importantly, Black youth with negative attitudes about their race experienced worse OCD symptomology, showing that internalized racism may affect OCD impairment [44]. Additionally, other studies found that Black youth endorse increased washing, cleaning, and contamination symptoms and suggested this may be affected by negative racial stereotypes [45,46].

In another example, Latiné youth engage with religion within a cultural and familial-based context, and this connection might lead to a misunderstanding from clinicians about the cultural value of religion vs. reports of distressing obsessions indicative of religious scrupulosity [47]. This is especially important to consider as religious scrupulosity is associated with worse mental health impairment than general religiosity for Latiné individuals. Additionally, as family opinions are incredibly important, religiosity might not be noticed or reported as becoming scrupulous. The role of religion should be considered when assessing for obsessions, as the stigma against mental illness in Latine families usually creates a dependence on religion and spirituality for coping.

There is a disparity that exists for Black and/or Latiné youth to mental health care access compared to White youth. Black and Latiné youth are the least likely to be represented in treatment utilization for OCD [42,43]. Similarly, Black and/or Latiné youth with ADHD are also often overlooked in diagnosis, treatment utilization, and medication usage [48,49]. The implication of structural and systemic racism from practitioners and researchers hinders the quality of life of Black and Latiné youth with ADHD and OCD. Both Black and Latiné youth are reported to have significant issues with social and academic performance due to complications with ADHD symptoms [49] and have lower income, employment instability, and educational prospects with limited care for their OCD symptoms as adults [39].

### 1.6. Present Study

The current literature supports that there is a potential connection between ADHD and OCD symptoms and that secondary psychological symptoms such as depression, anxiety, and trauma are associated with ADHD and OCD symptoms. In this present study, we examined the relationship between inattentive and hyperactivity/impulsive ADHD symptoms, obsessive-compulsive symptoms, and anxiety disorders for Black and/or Latiné youth residing in the United States. In addition, we examined how comorbid psychological conditions such as depression, anxiety, and trauma are associated with ADHD, OCD, GAD, social anxiety disorder, and panic disorder. Within the current literature, there is almost no research that has included Black and/or Latiné youth with ADHD. This study sought to rectify this limitation. Our research questions include, (1) what the associations between ADHD symptoms and obsessions and compulsions are and (2) how ADHD, OCD, and anxiety disorders are associated with depression, anxiety, developmental trauma, and adverse childhood experiences. We hypothesize that ADHD and OCD will have a positive relationship; specifically, among ADHD symptoms and obsessions. We also expect that depressive, anxious, and traumatic symptoms will be positively associated with both ADHD and OCD symptoms and anxiety disorders. Additionally, we expect ADHD and anxiety disorders to be positively associated with trauma symptoms.

## 2. Methods

### 2.1. Participants

63 Black and/or Latina/é/o youth were enrolled in a larger study funded by the Robert Wood Johnson Foundation that provided free psychodiagnostic assessments for Black and/or Latiné youth suspected of having difficulties with attention. ADHD was purposefully not mentioned on the flyers as there is stigma around seeking an ADHD diagnosis. This will be the first manuscript published from this study. Participants were recruited in the United States via local Chicagoland middle schools’ and high schools’ referrals, community centers, pediatric clinic referrals, flyer advertisement, and social media engagement. Participants are between the ages of 11–18 years old (6th to 12th grade) and were accompanied by their parents and/or legal guardians during the initial visit. All adolescent participants were Black, Latina/e/o, multicultural, and/or Indigenous.

The mean age of the participants was 14 years old (*M* = 14.44). The mean age of participants diagnosed with ADHD inattention presentation was 14 years old (*M* = 14.77), participants with ADHD combined presentation were 14 years old (*M* = 14.05), participants diagnosed with ADHD hyperactivity/impulsivity were 11 years old (*M* = 11.00), and participants with other specified ADHD were 15 years old (*M* = 15.50). 29.1% of participants were Black, 20.8% were Latiné, 12.5% were Black-Latiné, and the remaining 37.6% were Multicultural (more than one race, ethnicity, and/or cultural identity, see Table 1). The sample had slightly more cisgender boys (43.8%) with the remaining 41.7% cisgender girls, <10% as non-binary, and <10 being unsure of their gender identity. Overall, of the participants in this sample, 20.8% reported being LGBT+. Among the families, 32.6% had an annual household income between $40,000–60,000.

Table 2 shows the prevalence of conditions across studies. Kessler et al. [12] is an epidemiological sample, Arnold et al. [2] is a clinical sample, and Peles et al. [23] a cross-sectional clinical sample.

### 2.2. Procedures

Parents and/or legal guardians of adolescents completed a phone screening at initial contact to determine eligibility to enroll in the study. Participants who met the eligibility criteria during the phone screening were enrolled into the present study and invited for a 3-h psychodiagnostic assessment. Eligibility criteria included adolescent participant identifying as Black and/or Latiné, being in 6th–12th grade at the time of study entry, adolescent having no evidence of a severe developmental delay from genetic origins (e.g., Down Syndrome), and parent and/or legal guardian having custody/medical decision-making authority of the adolescent participant. Additional criteria were the endorsement of 4 or more ADHD inattentive symptoms during phone screening.

Comparison groups (e.g., youth without ADHD, White youth) were not included as this study was specifically focused on Black and/or Latiné youth with ADHD, whose experiences do not need to be compared to others to be assessed and valued. For at least a century, psychology has focused on predominantly White youth samples, ignoring and excluding other youth. Thus, this project specifically and only included Black and/or Latiné youth with ADHD. Participants who met eligibility criteria during the phone screening and were interested in participating had informed consent obtained from parents and/or guardians and assent obtained from adolescents. After consent and assent are obtained, adolescents and their parents are tasked to complete baseline questionnaires prior to scheduled visits about their mental health and life experiences. During the scheduled visit, participants and their parents received a culturally responsive and comprehensive psychodiagnostic semi-structured interview examining depression, anxiety, developmental trauma, ADHD, and obsessive-compulsive symptoms by a doctoral-level clinician. To make this a culturally responsive interview, advisory boards were created with parents and adolescents to provide feedback on how best to ask questions. Using this feedback, a qualitative interview was developed, including questions about identity, joys, and strengths while also asking about oppression-related trauma (e.g., racism). Most clinical interviews focus on an individual’s symptoms, but these focus on the systems that affect youth and their families in addition to individual symptomology.

Academic achievement and intelligence testing were conducted via the Wechsler Intelligence Scale for Children (WISC-V), Wechsler Individual Achievement Test (WIAT-III), and Wechsler Abbreviated Scale of Intelligence (WASI-II) to assess neurocognitive abilities affected by conditions such as learning disabilities and ADHD. Feedback reports with diagnoses for adolescent participants are provided in a password protected document. Follow-up surveys are provided monthly to assess feedback about study, adolescent depression and anxiety symptoms, mental health service acquisition, and adolescent well-being. Participants are compensated at the assessment visit, feedback visit, and for each follow-up survey completed by parents and their child. For this study, only baseline measures will be used as follow-up survey data are still being collected.

### 2.3. Measures

Demographics. Demographic information was collected through self-report for adolescents and parents. The following information was collected from the adolescent: race and ethnicity, age, caregiver’s education level, approximate annual household income, age of birthing parent at birth, medical history, grade level, school name, and any known learning disabilities. The following information was collected from the parent/legal guardian: child age, their relationship to child, parent race and ethnicity, caregiver’s education level, approximate annual household income, age of birthing parent at birth, medical history, grade level, school name, and any known learning disabilities.

NICHQ Vanderbilt Assessment Scales, Parent Informant. The Vanderbilt Assessment Scales is a DSM-V based self-report scale that consists of 55 items that assess symptoms through the following subscales: ADHD, Oppositional Defiant Disorder (ODD), Conduct Disorder (CD). All items are rated on a 4-point scale: 0 = *never*, 1 = *occasionally*, 2 = *often*, 3 = *very often*. ADHD presentations were assessed through items 1–18; Items 1 to 9 assess inattentive ADHD symptoms and items 10 to 18 assess hyperactive/impulsive ADHD symptoms. The Vanderbilt measure has strong psychometric properties and high internal consistency [50].

ADHD Self-Report Scale. The self-report measure used for adolescents was used to assess inattentive, hyperactivity/impulsive, and combined ADHD presentations. This 18-item measure is scored on a 4-point scale: 0 = *never*, 1 = *occasionally*, 2 = *often*, and 3 = *very often*. Items 1 to 9 measure inattentive ADHD symptoms and items 10 to 18 measure hyperactivity/impulsivity ADHD symptoms.

Patient Health Questionnaire-9 (PHQ-9). The PHQ-9 is a self-report 10-item rating scale to assess depressive symptoms and severity. The participant is asked if the depressive symptoms have bothered them over the last 2 weeks. Items 1 to 9 on the scale consist of questions answered on a 4-point Likert scale: 0 = *not at all*, 1 = *several days*, 2 = *more than half the days*, 3 = *nearly every day*. Item 10 asks if any symptoms that were answered with a 1 or higher lead to any difficulty for the participant in their work, school, home, or interpersonal relationships. Item 10 was also answered on a 4-point Likert scale: 0 = not difficult at all, 1 = somewhat difficult, 2 = very difficult, 3 = extremely difficult. The PHQ-9 is noted as a reliable clinical and research tool and with excellent internal reliability [51].

Generalized Anxiety Disorder 7-Item (GAD-7). GAD-7 is a self-report 7-item rating scale to assess anxiety symptoms and severity. The participant is asked if anxious symptoms have affected them in the past 2 weeks. Items 1 to 7 are answered on a 4-point Likert scale: 0 = *not at all*, 1 = *several days*, 2 = *more than half the days*, 3 = *nearly every day*. The GAD-7 has excellent internal reliability (0.92) and validity (0.83) and is an efficient tool to assess anxiety [52].

Adverse Childhood Experiences (ACEs) Questionnaire. ACE is a self-report 10-item rating scale administered during the initial in-person interview to assess psychological, physical, emotional, and sexual abusive experiences that might have occurred before the age of 18. The adolescent is asked to answer “yes” or “no” for these questions; “no” is scored as 0 and “yes” is scored as 1 [53].

Developmental Trauma Symptom Screening Checklist. The Developmental Trauma (DTD) Symptom Screening checklist is used to assess emotional regulation difficulties, social/interpersonal difficulties, behavioral difficulties, physiological difficulties, and cognitive difficulties produced after childhood trauma. The checklist is not a diagnostic tool but rather an endorsement of problem areas after an adolescent has experienced trauma during their developmental years. The initiative to consider a formal criterion for DTD can highlight the role of traumatization effects on developmental outcomes for youth and the comorbidity with neurodevelopmental conditions such as ADHD and OCD [54].

The Kiddie Schedule for Affective Disorders and Schizophrenia (K-SADS). K-SADS is a semi-structured diagnostic interview administered to parents and children to assess mental health diagnoses in youth ages 6–18. K-SADS assesses for an assortment of child psychiatric disorders including ADHD, OCD, depression, anxiety, and PTSD. K-SADS items are scored on a 0 to 3-point scale: 0 indicates no information is available, 1 indicates the symptom is not present, 2 indicates subthreshold levels of the symptom, and 3 indicates threshold criteria. When administered, parents and adolescents were interviewed separately. Interview time was typically around 40 min to complete for parent and adolescent. The K-SADS is considered a valid and reliable clinical interview for assessing psychiatric conditions among youth [55].

### 2.4. Data Analysis

All statistical analyses were performed in Mplus V.8. Statistical analysis was conducted with 48 participants after removing missing and/or incomplete data. Participants were included in statistical analysis if they had a confirmed ADHD diagnosis after initial in-person assessment, adolescent completed baseline survey measures, and parent and adolescent completed KSADS interview. 15 participants were excluded from the analysis due to participants not having ADHD and/or missing or incomplete measures from adolescents or parent/legal guardians. 4 participants that had missing parental/guardian survey data remained due to parents completing the KSADs interview. KSADs interview was used to collect reported symptoms from adolescents and parents/legal guardians for all conditions. In addition, all other symptoms other than OCD and anxiety diagnoses were collected via survey: inattentive and hyperactive/impulsive ADHD symptoms were calculated using self-reported and parent-reported ADHD symptoms; depressive symptoms were calculated from PHQ-9; anxiety symptoms were calculated from GAD-7; and trauma symptoms were reported from ACEs and DTD checklists. 

Obsessions and compulsions were calculated from subthreshold and threshold data collected from the KSADs. Types of obsessions and compulsions were also calculated from subthreshold and threshold data reported from participants and parents.

A frequency table was created to assess the most endorsed obsessions and compulsions. Bivariate correlations were examined to determine the associations between each variable. Next, a path analysis, which accounts for all associations within the model, was run to assess the strength of the relationship between inattentive and hyperactive/impulsive ADHD symptoms as predictor variables with obsessions and compulsions as outcome variables. Finally, another path analysis was run to assess the relationship between inattentive ADHD symptoms, hyperactive/impulsive ADHD symptoms, obsessions, compulsions, and anxiety disorders as predictors of depression, anxiety, and trauma.

## 3. Results

The total prevalence rate included 54.2% of participants meeting criteria for ADHD inattentive presentation (*N* = 26), 39.6% meeting criteria for ADHD combined presentation (*N* = 19), 2.1% meeting for ADHD hyperactive presentation (*N* = 1), and 5% (*N* = 2) of participants met criteria for other specified ADHD. The prevalence rate for an OCD diagnosis in our sample is 6.8% (*N* = 3); a participant only diagnosed with OCD and not ADHD was excluded from statistical analysis, but including this participant overall prevalence is 8.3% (*N* = 4). See Table 2 for prevalence rate information. Regardless of diagnosis, multiple participants reported clinically significant levels of obsessions and compulsions that impaired them in daily life. 8.3% (*N* = 4) of participants reported they were currently having subthreshold compulsions and obsessions. 2.1% (*N* = 1) reported experiencing current threshold compulsions and 4.2% (*N* = 2) reported experiencing current threshold obsessions. 4.2% (*N* = 2) reported having subthreshold obsessions and 2.1% (*N* = 1) reported having threshold obsessions in the past. 4.2% (*N* = 2) parents reported their child having subthreshold compulsions and 2.1% (*N* = 1) reported their child experiencing threshold compulsions. 4.2% (*N* = 2) of parents only reported threshold level obsessions from the participants currently and in the past. 31.25% (*N* = 15) of participants were diagnosed with an anxiety disorder, including social anxiety disorder and generalized anxiety disorder.

Table 3 illustrates the frequency of current compulsions and obsessions reported by participants and their parents during the KSADs interview. Common compulsions reported by participants include touching (*N* = 2), counting (*N* = 2), cleaning and washing (*N* = 2), checking (*N* = 2) and repeating (*N* = 3). Common obsessions reported by participants included morbid thoughts (*N* = 2), need for symmetry (*N* = 2), and meaningless phrases (*N* = 2). Parent-reported obsessions and compulsions did not have much frequency, but parents overall reported more obsessions than compulsions. Interestingly, the only parent-reported compulsion was repeating (*N* = 1).

A bivariate correlation analysis revealed several significant relationships among study variables shown in Table 4. Interestingly, compulsive symptoms did not show significant correlations with any of the mental health outcomes: trauma (−0.04), anxiety (0.03), and depression (0.04).

**Figure 1:**
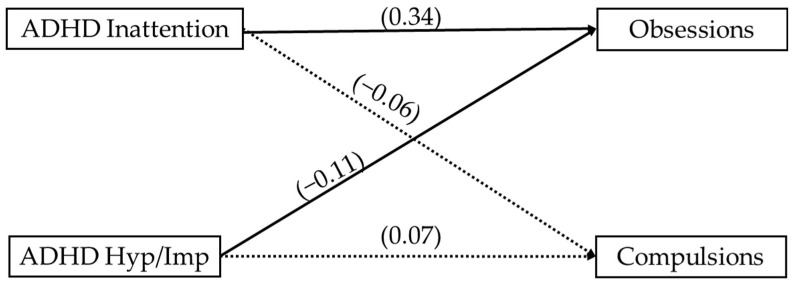
Path analysis examining associations between ADHD presentations and OCD symptoms.

A path analysis examining the relationship between ADHD symptoms and OCD obsessions and compulsions is illustrated in Figure 1. We found that ADHD inattentive symptoms were positively associated with obsessions (β = 0.34. *p* < 0.01) and ADHD hyperactive/impulsive symptoms were negatively associated with obsessions. (β = −0.11, *p* < 0.05). Neither ADHD inattention nor ADHD hyperactive/impulsive was significantly associated with compulsions, which aligned with bivariate associations.

**Figure 2:**
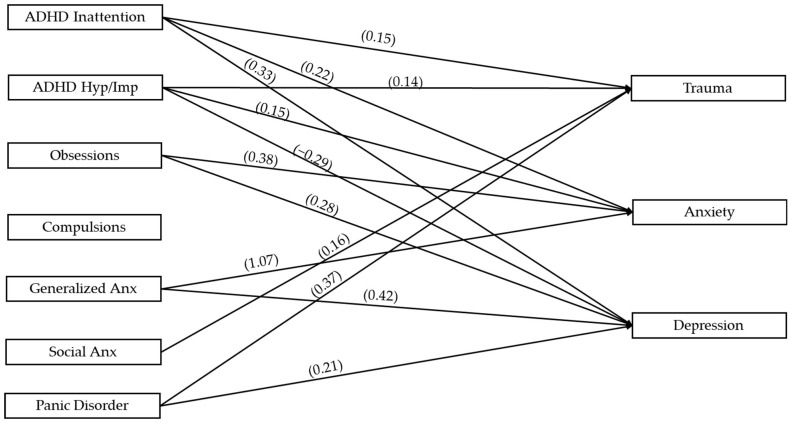
Path analysis examining associations between variables of interest.

Figure 2 illustrates the path analysis with all variables of interest. We found that ADHD inattention was positively associated with trauma (β = 0.15, *p* < 0.05), anxiety (β = 0.22, *p* < 0.01), and depression (β = 0.33, *p* < 0.05). ADHD hyperactive/impulsive symptoms were negatively associated with depression (β = −0.29, *p* < 0.01) and positively associated with trauma (β = 0.14, *p* < 0.05) and anxiety (β = 0.15, *p* < 0.05). Obsessions were positively associated with anxiety (β = 0.38, *p* < 0.001) and depression (β = 0.28, *p* < 0.01). Compulsions were not associated with any outcome variables. GAD was positively associated with anxiety (β = 1.07, *p* < 0.001) and depression (β = 0.42, *p* < 0.001), panic disorder was associated with depression (β = 0.21, *p* < 0.01) and trauma (β = 0.37, *p* < 0.001), and social anxiety disorder was only associated with trauma (β = 0.16, *p* < 0.05; see Table 5).

## 4. Discussion

The present study was conducted to evaluate the association between obsessive and compulsive symptoms with ADHD inattentive and hyperactive/impulsive symptoms for Black and/or Latiné youth. In addition, this study examined the connection between OCD, ADHD, and anxiety disorders with other commonly comorbid symptomology—depression and anxiety, and trauma exposure. Importantly, this study included adolescents who were Black and/or Latina/é/o and several identified as LGBTQ+, who are often excluded from this research. The clinical and empirical implications of these findings can assist in tailoring and supporting adolescents and their families in the future. As this study was conducted in the United States, these findings should be considered within the cultural context of the systemic racism and prejudice that Black and/or Latiné youth experience.

The prevalence of OCD diagnosis in this sample was slightly lower than expected as only 6.8% of eligible participants met criteria; however, this remains relatively consistent with literature based on prevalence of OCD for people with ADHD (~11%) [2,13]. Importantly, this is a small sample size and prevalence must be replicated in a larger sample of Black and/or Latiné youth with ADHD. Regardless of diagnostic prevalence, 10.4% of participants reported experiencing clinically significant compulsions and 12.5% reported experiencing clinically significant obsessions. This illustrates an association between compulsions and/or obsessions among adolescents with ADHD. Somewhat consistent with our hypotheses, obsessions were associated positively with inattentive symptoms (β = 0.34) and negatively with hyperactivity/impulsivity (β = −0.11). Obsessions were also positively associated with anxiety (β = 0.38) and depression (β = 0.28) symptomology. Compulsions were completely unrelated to other variables in this study. The associations between internalizing symptoms, such as anxiety and depression with obsessions were expected, especially as OCD was once classified as an anxiety disorder and obsessive symptoms are a major predictor of reported depression [25,28,56]. Also, while unexpected, the negative association between obsessions and hyperactivity/impulsivity symptoms remains in line with the literature that suggests that OCD and ADHD hyperactivity/impulsivity symptomology sit opposite on a compulsive-impulsive continuum [1,14,15]. As hyperactivity-impulsivity symptoms are different in presentation, these results could potentially highlight that obsessive-compulsive symptoms are more related with people with ADHD inattentive presentation. However, as hyperactivity-impulsive and inattention symptoms are connected to anxiety, there could be an implication that generally, ADHD symptoms invoke greater reports of anxiety.

Common Obsessions/Compulsions. There was no clear pattern found within our sample in terms of obsessions and compulsions endorsed. Obsessions that were most frequently reported included morbid thoughts (*N* = 2), need for symmetry (*N* = 2), and meaningless phrases (*N* = 2). The compulsions that had the highest reported frequency were touching (*N* = 2), cleaning and washing (*N* = 2), checking (*N* = 2), and repeating (*N* = 3). Although there is no obvious pattern, the compulsions are ones focused on executive functioning skills like organizing (checking) and working memory (repeating). Adolescents with ADHD may be more inclined to check things repeatedly due to forgetting items or tasks in the past, leading to a secondary diagnosis of OCD to try to account for their ADHD symptoms. Often, adolescents with ADHD experience punishment for forgetting or losing items due to the cost incurred to replace items [57]. They are also punished in school for making mistakes by being told they are not trying hard enough or earning poor grades, which may increase checking behaviors to decrease this punishment [58]. There also may be underreporting of these obsessions and/or compulsions by both adolescents and caregivers due to a lack of information on what these symptoms might look and feel like, or other cultural factors. Although religious scrupulosity is often attributed to Black and Latiné youth as a common compulsion, none were reported by the adolescents and parents in this study. The clinical implications could be that there is a generational shift in perspectives of religion and spirituality, or that current diagnosis tools have little cultural competency to recognize nuance in healthy and unhealthy religious thoughts reported by Black and/or Latiné youth. Generally, there is a need for more investigations into how obsessions and compulsions are reported by Black and Latiné youth and families, and culturally responsive assessments are beneficial to finding distinctions for heterogeneous expressions of obsessions. This must be done in a larger, more representative sample of Black and/or Latiné youth with ADHD.

It is important to note that obsessions and compulsions were not significantly correlated in this sample. Additionally, compulsions were not significantly associated with any variable of interest, while obsessions were associated with anxiety and depression. These findings may be influenced by the specific types of obsessions and compulsions reported. Compulsions that are more behaviorally driven may not elicit the same level of emotional distress as intrusive obsessions related to harm or contamination, which are more cognitively distressing. As a result, the lack of association between compulsions and internalizing symptoms suggests that compulsions alone may not be a reliable indicator of anxiety or depression in Black and/or Latiné youth with ADHD. This distinction highlights the need for clinicians to assess the broader context of OCD symptoms rather than assuming compulsions contribute equally to internalizing distress. Future research should explore whether specific types of obsessions and compulsions interact differently with ADHD and internalizing symptoms, which could inform more targeted and effective clinical interventions. Obsessions may also be more common than compulsions for Black and/or Latiné adolescents with ADHD due to increased emotion dysregulation, inattention, or becoming hyper-fixated on a certain thought or thought pattern [59,60]. As many youth with ADHD report difficulty with identifying thoughts, it could be that OCD is used to correct this difficulty by focusing on a thought too much, leading to impairment [61,62].

Obsessions were only associated with depression and anxiety, not trauma. Our sample had high rates of trauma, with 100% (*N* = 48) of participants reporting some trauma experience in their lives. This could mean our sample lacked enough heterogeneity to truly understand how OCD and trauma are associated, as the prevalence of OCD was relatively low. In addition, it could be that trauma and OCD are just not associated with each other for Black and/or Latiné youth with ADHD, but a larger sample is needed to discern this connection.

Inattention and Hyperactivity/Impulsivity. Hyperactivity and impulsive symptoms were negatively correlated with obsessions (β = −0.11) and depression (β = −0.29) and positively associated with trauma (β = 0.14) and anxiety (β = 0.15), while inattentive symptoms were positively associated with obsessions (β = 0.34), trauma (β = 0.12), anxiety (β = 0.13), and depression (β = 0.22). Although somewhat unexpected, there are a few potential reasons as to why higher rates of hyperactivity/impulsivity are associated with fewer obsessions and less depression. For instance, hyperactivity and impulsiveness make people restless, which may decrease withdrawn behaviors that are often associated with depression or the amount of time spent on an obsessive thought. It is possible that restlessness and impulses are protective of increasing depressive symptoms when accounting for OCD. Additionally, parents of high-energy adolescents may provide more active opportunities to decrease their restlessness (e.g., engagement in sports, active extracurriculars) that may belie depressive symptoms. Another potential explanation for this association may point to difficulties with executive functioning or challenges focusing on racing thoughts for those who endorse hyperactivity and impulsive symptoms, contributing to less rumination on distressing thoughts or obsessions. Additionally, the internalizing nature of both obsessions and depression in terms of behavior patterns, thoughts, and coping strategies may explain this inverse relationship with externalizing hyperactivity/impulsivity symptoms in terms of behavior. Interestingly, the more hyperactive/impulsive symptoms reported, the higher the rate of current anxiety symptoms and past trauma exposure. Anxiety behavior might manifest as fidgeting, restlessness, and excessive talking for youth who exhibit hyperactivity and look very similar to hyperactive/impulsive symptomology of ADHD. Considering that Black and/or Latiné also receive increased punishment and supervision in school for “acting out” or hyperactive and impulsive behaviors, it is no surprise that this would increase their anxiety and trauma response. For example, a Black and/or Latiné adolescent who gets in trouble for hyperactive/impulsive behaviors in the classroom sees their White peers being treated differently, raising their awareness of inequitable treatment (i.e., racism), which understandably increases anxiety. In addition, youth who are hyperactive or impulsive may be more likely to find themselves in dangerous situations, leading to higher exposure of trauma [36].

The more a Black and/or Latiné adolescent with ADHD expresses inattention, the higher rates of trauma exposure and anxiety and depressive symptoms they experience. With ADHD and trauma, it is often a circular nature, with youth with ADHD experiencing more trauma exposure and a lack of a neurodiverse safe world leads to not paying attention or noticing certain unsafe patterns that lead to trauma exposure [36]. As ADHD typically begins in childhood, the usually lifelong complications that arise from inability to concentrate can create academic underperformance, unstable interpersonal connections, and low self-esteem, which might lead to depressive and anxiety symptoms [63,64]. When accounting for OCD, inattention and depression might lead to more obsessions due to the internalizing nature of all three symptoms. More obsessive thoughts could occur, stemming from an inability to concentrate on other tasks or engage in the world around them. In addition, inattentive ADHD symptoms could be exacerbated by increased depression and obsessions, leading to more difficulties with concentration and their working memory and executive functioning. Importantly, Black and/or Latiné youth exist within family, community, and school systems that often provide negative feedback to disorganization and impulsivity, potentially creating a reinforced feedback loop to increase organization or perfectionistic thinking. They may be rewarded by family members and teachers for higher levels of control, leading to more obsessions and compulsions for organization.

Anxiety Disorders. As ADHD symptoms can be overwhelming and impairing in daily life, the prevalence of moderate anxiety symptoms was expected with ~31% of participants meeting criteria for an anxiety disorder. Overall, each anxiety diagnosis was associated with current mental health experiences of anxiety or depression or past trauma exposure. GAD had the strongest associations with outcomes, with expectedly high rates of current anxiety and depression symptomology. GAD often co-occurs with depressive disorders [3], and although not in the scope of this study, it is likely that youth with GAD had high rates of major depressive disorder or persistent depressive disorder. Youth with GAD are often characterized as “worriers”, which may lead them, despite their ADHD, to attend to their world at higher rates, leading to lower likelihood of trauma. This hypervigilance or over awareness of their environment may also be due to heightened punishment Black and/or Latiné youth experience in communities and school, making these youth have higher worries about experiencing additional trauma and trying to avoid or disengage when these situations occur.

Social anxiety and panic disorders were both associated with higher rates of trauma exposure, which might be related to mismanaged or misunderstood ADHD symptoms. This often leads to poor relationships with peers and psychosomatic responses to perceived stressful situations [65]. Importantly, approximately 86% of youth in this sample received a first time ADHD diagnosis, so a majority of the youth in this study were unaware of their diagnosis and were not receiving support or treatment for their difficulties with attention and hyperactivity/impulsivity. In addition, the connection that these diagnoses display with depression could be related to the inattentive symptoms expressed, which leads to more internalization of the world around them and shame associated with neurodiversity due to ableism [66]. Often, youth with ADHD experience punishment for merely existing with ADHD, leading to high rates of depression and anxiety [67].

Treatment strategies. Black and/or Latiné youth with ADHD, OCD, and anxiety disorders are experiencing higher rates of trauma, depression, and anxiety, thus, medication and therapy are likely an important option for these adolescents. Unfortunately, there are only a few studies that examined treatment effectiveness for comorbid ADHD and OCD. As OCD and ADHD have opposing differences in frontostriatal functioning, it is difficult to determine medication for co-morbid ADHD and OCD [1]. Stimulant medication, part of first line treatment for ADHD, may exacerbate OCD symptoms as well as comorbid trauma reactions and anxiety [59]. As such, it is suggested that co-morbid OCD, anxiety, and ADHD include using serotonin reuptake inhibitors (SSRIs) and CBT and exposure prevention response (ERP), not stimulant medication [1,59]. Even though SSRIs and CBT/ERP are first line treatment for OCD and not ADHD, they have been found to improve attention symptoms. Comorbid ADHD and OCD had a diminished treatment response to CBT, perhaps attributed to experiencing greater impairment with executive functioning and a greater likelihood of other comorbidities than individuals with only OCD or ADHD [68]. Unfortunately, most treatment providers are not well-versed in how to support neurodiverse youth, which is also partially to blame for this diminished treatment response. Multimodal treatment strategies that integrate behavioral interventions, medication management, and family involvement are crucial for addressing the complex interactions between these diagnoses. Early intervention and a holistic treatment plan can help mitigate the long-term effects of these comorbid conditions and improve overall functioning [1].

Despite the prevalence and impact of these co-occurring disorders, research on ADHD and OCD for Black and/or Latiné youth is limited. Existing studies generally focus on these disorders separately, with little exploration of their combined effects. Black and Latiné youth with ADHD, for example, are less likely to receive adequate treatment compared to their White peers, which may contribute to more severe outcomes [33]. Cultural stigma surrounding mental health and racism in the medical and psychological fields also prevents families from seeking treatment, leading to underdiagnosis and undertreatment of OCD in youth with ADHD. Black young people who experienced racial discrimination report higher distress from the OCD symptoms, suggesting the need for racism-based therapy options to support Black and/or Latiné youth with OCD [35]. Thus, a combination of SSRIs, CBT/ERP, and culturally responsive therapy is warranted. One example is EMBRace, an intervention to reduce racial stress and enhance racial coping by providing validation for experiences of racism, providing support, preparing for bias, and increasing connection and coping [69]. Likely, providers could incorporate EMBRace with ERP/CBT, behavioral management, and/or medication. Providers could also be more culturally responsive within ERP and CBT methodology, incorporating the understanding of the inequitable feedback loop Black and/or Latiné youth with ADHD experience, inclusive of racism and ableism in addition to other identities that may be oppressed. Finally, simply validating traumatic experiences, particularly oppression-related ones, goes a long way in being culturally responsive and supporting Black and/or Latiné youth regardless of ADHD and OCD.

### Limitations

This study has several limitations that should be considered. First, the sample primarily consisted of individuals with inattentive and combined ADHD presentations, with fewer participants endorsing hyperactive/impulsive symptoms. This imbalance may have influenced the observed associations between ADHD and OCD symptoms. Second, although the KSADS semi-structured interview was administered to assess multiple mental health diagnoses, the primary focus was on ADHD, meaning OCD symptoms may not have been explored in as much depth, potentially leading to underreporting. We did not collect data on distress or impairment of obsessions and compulsions, which is needed to more comprehensively understand how OCD affects the lives of youth with ADHD. This may also be why compulsions were not associated with variables of interest in this study and if distress related to compulsions was assessed more thoroughly, results may differ. Moreover, as our sample consists of youth seeking psychodiagnostic assessments, this sample should not be considered an epidemiological representation of OCD and ADHD. Also, the sample was conducted in the United States and consisted entirely of individuals that lived in the Chicagoland area, limiting generalizability. Finally, the cross-sectional nature of this study limits the ability to determine causal relationships between ADHD, OCD, anxiety disorders, and internalizing symptoms. Future research should incorporate longitudinal designs to better understand how these symptoms develop and interact over time.

## 5. Conclusions

Black and/or Latiné youth with ADHD show higher rates of OCD and anxiety disorders than the general population. Overall, they experience high rates of anxiety, depression, and trauma, but no intervention to date has been developed to support the comorbidity of ADHD and OCD, nor the intersectionality of identity for Black and/or Latiné youth.

## Figures and Tables

**Table 1:** Demographic information.

Demographics	*N*	%
Racial/Ethnic Cultural Identity		
Multicultural	24	50.1
Asian & Black	<10	<10
Black & Latiné	6	12.5
Indigenous, Black, Latiné	<10	<10
Indigenous & Latiné	<10	<10
White & Latiné	13	27.1
Black	14	29.1
Latiné	10	20.8
Gender Identity		
Boy	21	43.8
Girl	20	41.7
Nonbinary	<10	<10
Unsure	<10	<10
Sexuality		
Straight	32	66.7
Lesbian	<10	<10
Pansexual	<10	<10
Queer	<10	<10
Not Sure/Questioning	<10	<10
Other	<10	<10
Don’t want to answer	<10	12.5
Medication Usage		
ADHD Medication	4	6.3
Emotional/behavioral Medication (e.g., SSRIs)	5	7.8
Sleep Medication (Melatonin only)	2	3.1
Smoking (e.g., e-cigarettes)	0	0

**Table 2:** ADHD, OCD, and comorbidity comparison across studies.

	CRAFT	Kessler [12]	Arnold [2]	Peles [23]
ADHD	93.7 (*n* = 45)	4.4%	88.8% (*n* = 134)	33.1% (*n* = 51)
OCD	2.1% (*n* = 1)	-	-	27.9% (*n* = 29)
Comorbid OCD and ADHD	6.8% (*n* = 3)	-	11.2% (*n* = 15)	16.9% (*n* = 26)

**Table 3:** Frequency of self-reported and parent-reported compulsions and obsessions.

Variable	Type	Self-Report Frequency	Parent-Report Frequency	Percent
Compulsions	Touch	2	0	4.2
	Counting	2	0	4.2
	Washing	2	0	4.2
	Checking	2	0	4.2
	Hoarding	1	0	2.1
	Ordering	1	0	2.1
	Scheduling	1	0	2.1
	Redo/Repeating	3	1	8.3
Obsessions	Contamination	1	0	2.1
	Aggressive Thoughts	0	1	2.1
	Morbid Thoughts	2	1	6.3
	Symmetry Need	2	0	4.2
	Meaningless Phrases	2	1	6.3
	Sexual Obsessions	1	1	4.2
	Hoarding/Saving	1	1	4.2
	Other	1	1	4.2

**Table 4:** Correlations between variables of interest.

Variables	IA	HI	Obsess	Compul.	Trauma	Anxiety	Depress.
Inattention	--						
Hyp/Imp	0.63 ***	--					
Obsessions	0.26 **	0.09	--				
Compulsions	−0.03	0.04	0.14 *	--			
Trauma	0.12 *	0.05	0.06	−0.04	--		
Anxiety	0.13 *	0.01	0.39 ***	0.03	0.16 *	--	
Depression	0.22 **	−0.06	0.34 ***	0.04	0.53 ***	0.52 ***	--

Note. * *p* < 0.05, ** *p* < 0.01, *** *p* < 0.001, Hyp/Imp = hyperactive impulsive, IA = inattention, HI = hyperactive/impulsive, Obsess = obsessions, Compul. = compulsions, Depress. = depression.

**Table 5:** Standardized Beta Coefficients for Full Path Analysis.

Variable	Trauma	Anxiety	Depression
	β		
ADHD Inattention	0.15 *	0.22 **	0.33 **
ADHD Hyperactive/Impulsivity	0.14 *	0.15 *	−0.29 **
Obsessions	0.06	0.38 ***	0.28 **
Compulsions	−0.06	−0.10	0.08
Generalized Anxiety Disorder	0.02	1.07 ***	0.42 ***
Social Anxiety Disorder	0.16 *	0.03	0.08
Panic Disorder Symptoms	0.37 ***	0.08	0.21 **

Note. * *p* < 0.05, ** *p* < 0.01, *** *p* < 0.001.

## Data Availability

Deidentified data and question text that directly pertain to the analyses included in this manuscript are available upon request.
